# Inhibitors of Urokinase Type Plasminogen Activator and Cytostatic Activity from Crude Plants Extracts

**DOI:** 10.3390/molecules18088945

**Published:** 2013-07-26

**Authors:** Xueqiang Zha, Ricardo Diaz, Jose Javier Rosado Franco, Veronica Forbes Sanchez, Ezio Fasoli, Gabriel Barletta, Augusto Carvajal, Vibha Bansal

**Affiliations:** 1 Department of Chemistry, University of Puerto Rico at Cayey, 205 Ave. Antonio R. Barcelo, Cayey, PR 00736, USA; 2 Department of Chemistry, University of Puerto Rico at Humacao, CUH Station, Humacao, PR 00792, USA; 3 Institute for Functional Nanomaterials, University of Puerto Rico, Facundo Bueso Bldg., San Juan, PR 00931, USA; 4 Department of Biology, University of Puerto Rico at Cayey, 205 Ave. Antonio R. Barcelo, Cayey, PR 00736, USA

**Keywords:** urokinase type plasminogen activator (uPA), uPA inhibitor, fibrin plate assay, *Croton lucidus*, metastasis, cytostatic activity

## Abstract

In view of the clear evidence that urokinase type plasminogen activator (uPA) plays an important role in the processes of tumor cell metastasis, aortic aneurysm, and multiple sclerosis, it has become a target of choice for pharmacological intervention. The goal of this study was thus to determine the presence of inhibitors of uPA in plants known traditionally for their anti-tumor properties. Crude methanol extracts were prepared from the leaves of plants (14) collected from the subtropical dry forest (Guanica, Puerto Rico), and tested for the presence of inhibitors of uPA using the fibrin plate assay. The extracts that tested positive (6) were then partitioned with petroleum ether, chloroform, ethyl acetate and *n*-butanol, in a sequential manner. The resulting fractions were then tested again using the fibrin plate assay. Extracts from leaves of *Croton lucidus* (*C. lucidus*) showed the presence of a strong uPA inhibitory activity. Serial dilutions of these *C. lucidus* partitions were performed to determine the uPA inhibition IC_50_ values. The chloroform extract showed the lowest IC_50_ value (3.52 µg/mL) and hence contained the most potent uPA inhibitor. Further investigations revealed that the crude methanol extract and its chloroform and *n*-butanol partitions did not significantly inhibit closely related proteases such as the tissue type plasminogen activator (tPA) and plasmin, indicating their selectivity for uPA, and hence superior potential for medicinal use with fewer side effects. In a further evaluation of their therapeutic potential for prevention of cancer metastasis, the *C. lucidus* extracts displayed cytostatic activity against human pancreatic carcinoma (PaCa-2) cells, as determined through an MTS assay. The cytostatic activities recorded for each of the partitions correlated with their relative uPA inhibitory activities. There are no existing reports of uPA inhibitors being present in any of the plants reported in this study.

## 1. Introduction

There is abundant experimental evidence that urokinase-type plasminogen activator (uPA) is a multifunctional serine protease which possesses mitogenic, chemotactic, adhesive and migratory properties and thus plays an essential role in the process of tumor cell metastasis, aortic aneurysm, and multiple sclerosis [1,2,3,4,5,6,7,8]. The mechanism of uPA function involves binding to its receptor (uPAR) thus initiating a proteolytic cascade that results in the conversion of plasminogen to plasmin. While this formation of plasmin and its subsequent role in thrombolysis have been studied in great details, there is also an increasing interest in its role in the induction of cancer cell migration. The plasmin generated as a consequence of uPA activity is known to activate certain matrix metallo-proteases (proMMPs) thereby triggering extracellular matrix degradation leading to tumor cell metastasis [8]. Furthermore, metastatic cancer cells are marked by an over expression of uPA or uPAR [9]. Therefore, uPA is an attractive target for anti-invasiveness. As the uPA system has been shown to be non-essential for animal development or fertility [10], the inhibition of uPA activity could lead to prevention of metastasis with fewer side effects. Undisputed clinical data in fact exist in support of the uPA system being a promising target for novel tumor biological therapy [7]. Strategies that target uPA system include antisense technology, monoclonal antibodies, cytotoxic antibiotics (targeting uPA receptor), and synthetic inhibitors of uPA [2]. Since antiproteolytic therapy through enzyme inhibition, is one of the preferred strategies for cancer treatment, the uPA system is the obvious choice for manipulation [11,12]. Inhibitors of uPA are thus good candidates for use as drugs in treatment of cancer and also other disease situations where uPA-driven degradation of extra cellular matrix or uPA-dependent cell migration is thought to be important [13].

At least two inhibitors of uPA: *para*-aminobenzamidine [11] and amiloride [12], have been shown to reduce tumor growth in animal models. The potential use of other anitifibrinolytic drugs for this purpose, such as aprotinin and tranexamic acid, has also been studied and discussed [5]. A leupeptin analogue inhibiting the uPA-plasmin system was also found to suppress *in vitro* invasion of human fibrosarcoma cells [14]. However, none of these known inhibitors of uPA are likely to be used in anticancer therapy because of their weak inhibitory activity or high toxicity [7].

Natural compounds are preferred for chemoprevention for a variety of reasons that include: ease of oral application, regulatory approval, mechanism of action, and most importantly, potential human acceptance [7,15]. Inhibitors of uPA are reportedly present in many plant products. Fan *et al*. reported that popular fruits (kiwi), vegetables (peas, spinach, pumpkin, lemon), and tea consumed in daily life could inhibit uPA activity and may be helpful in the prevention of malignant tumor formation [16]. Inhibitors obtained from plants offer the additional advantage of their possible use as nutraceuticals. It has been said that a proper diet rich in uPA-inhibiting nutraceuticals might support the prevention of prostate cancer and be a supportive tool in prostate cancer treatment [17]. The presence of inhibitors of uPA in some forage crop species has also been reported [18]. Chinese herbs have also been studied in this regard. For example, *Selaginella* *tamariscina* extracts have been found to contain metastasis in lung carcinoma cells by causing decreased expression of uPA [19]. The anti-metastatic and anti-angiogenic activity of *P. angulata*, a well-known Chinese medicinal herb, has also been demonstrated as a consequence of its ability to inhibit uPA [4]. In another such study, Ishii *et al*. found that the extract from *Serenoa repens* suppressed the invasion activity of human urological cancer cells by inhibiting uPA without affecting the viability, adhesion ability, or motility of the cell lines [6].

While there are innumerable reports on screening of plants from different regions of the World for cytotoxic activity, there are very few that actually target uPA, some of which have been described above. There is thus a vast scope for discovery of new inhibitors of uPA that could provide superior alternatives to current drug candidates for prevention of cancer metastasis.

Puerto Rico is a tropical island, home to a huge floral diversity ranging from rain forest vegetation to a dry forest flora. The goal of this study was to screen plants from Puerto Rico, traditionally known for their medicinal (mostly antitumor) value, for the presence of inhibitors of uPA. It is well known that aqueous and ethanolic extracts from plants used in allopathic medicine are potential sources of antiviral and antitumor agents [20]. The crude plant extracts were thus prepared in methanol and screened for inhibitors of uPA using assay specific for uPA activity. The most promising extract was then partitioned with solvents of different polarities and investigated further for its uPA inhibitory and cytostatic properties.

## 2. Results and Discussion

Plants are treasuries of secondary metabolites having various structures and biological activities. As a consequence, at least 25% of our pharmaceuticals have plant origins [21]. Thus, a place such as the tropical island of Puerto Rico, with its rich biodiversity spanning from rain forest vegetation (El Yunque) to subtropical dry forest (Guanica) flora, offers immense potential for the discovery of new drug candidates. Puerto Rico has over 135 plants with recognized major medicinal uses and an additional 170 of minor therapeutic value [22]. However, apart from the use in folk medicine, this tropical wealth has largely escaped exploration for plant based drug discovery. In this study, we have screened a selection of plants from Puerto Rico, known traditionally for their medicinal value, for presence of inhibitors of uPA. This class of inhibitors is of extreme medicinal importance due to the aforementioned implication of uPA in cancer metastasis and related disorders.

### 2.1. Screening of Medicinal Plants for uPA Inhibitory Activity

Fourteen plants were collected from the subtropical dry forest at Guanica, Puerto Rico, based on their traditional use for medicinal purposes. The crude methanol extracts of the leaves of these plants were prepared and tested for presence of inhibitors of uPA by fibrin plate assay (Table 1). Among the 14 plants, uPA inhibition was observed in six species including *Croton lucidus*, *Erythroxylum aerolatum*, *Tabebuia heterophylla*, *Lantana camara*, *Cananga odorata*, and *Amyris elemifera*.

**Table 1:** Screening of plant extracts for inhibition of uPA.

Plant species	Origin	uPA inhibitory activity *
*Amyris elemifera*	Florida, Bahamas, Puerto Rico, West Indies, Central America.	+
*Bidens alba L.*	Native to South America. Pan tropical weed	–
*Cananga odorata*	Native to southern Asia and Philippines	+
*Capparis cynophallophora*	Florida, Bahamas, Puerto Rico, West Indies, Central America.	–
*Colubrina elliptica *	Puerto Rico, Florida, Bahamas, West Indies, Central Mexico toVenezuela	–
*Croton lucidus *	Native to PR, Bahamas, Greater Antilles, and Grand Cayman.	+
*Erythroxylum areolatum *	Bahamas, Greater Antilles, and South America	+
*Exostema caribaeum *	Florida, Bahamas, West Indies, Mexico, Central America, NE coast South America	–
*Guaiacum officinale *	Continental Tropical America, PR, West Indies	–
*Kalanchoe pinnata *	Native to Madagascar	–
*Lantana camara *	Pantropical	+
*Plumeria alba L. *	Central America and the Caribbean	–
*Tabebuia heterophylla *	Puerto Rico, Hispaniola, Virgin Islands, and Lesser Antilles.	+
*Vanilla claviculata *	Puerto Rico and Greater Antilles	–

* + indicates positive uPA inhibition activity; – indicates absence of uPA inhibition activity.

The plants that tested positive for uPA inhibitory activity were then partitioned with organic solvents of different polarities (petroleum ether, *n*-butanol, chloroform, ethyl acetate), dried and resuspended in methanol, and each partition tested for the presence of inhibitors of uPA (Table 2). uPA inhibitory effects occurred in the ethyl acetate extracts of four plants, chloroform extracts of two plants, *n*-butanol extracts of two plants, and petroleum ether extract of only one plant. Among the six active plants, *Croton lucidus* (*C. lucidus*) showed the presence of uPA inhibitors in all four partitions, with the chloroform partition causing the strongest inhibition as evidenced by the size of lysis zones on the fibrin plates. Based on these results, further experiments were carried out with the *C. lucidus* extract partitions to determine the IC_50_ values and selectivity of the uPA inhibitors present in the partitioned extracts.

**Table 2:** Screening of plant extract partitions for inhibition of uPA.

Plant name	Partitions from crude methanol plant extracts *			
	Petroleum ether	Chloroform	Ethyl acetate	*n*-Butanol
*Amyris elemifera*	–	–	+	–
*Cananga odorata*	–	–	+	–
*Croton lucidus*	+	+	+	+
*Erythroxylum areolatum*	–	–	–	+
*Lantana camara*	–	+	–	–
*Tabebuia heterophylla *	–	–	+	–

* + indicates positive uPA inhibition activity; – indicates absence of uPA inhibition activity.

### 2.2. uPA Inhibitory Activity of C. lucidus Extracts

**Figure 1:**
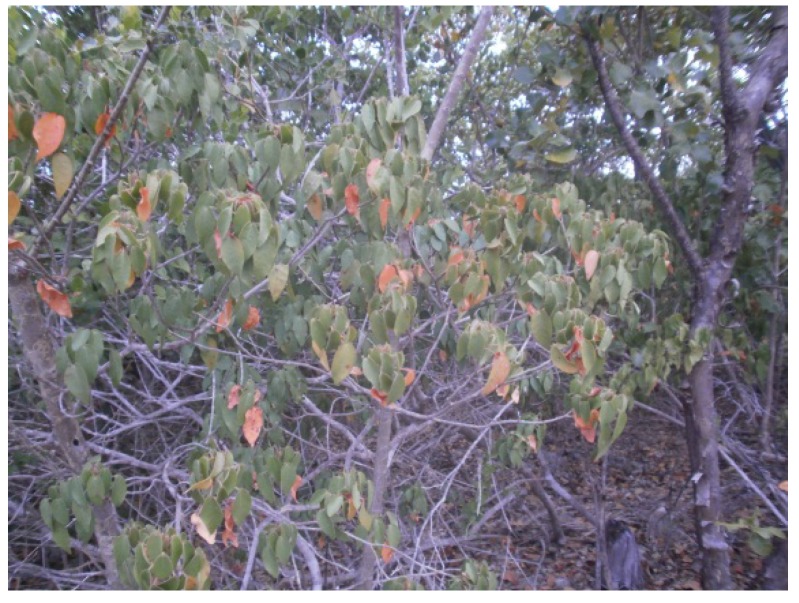
*C. lucidus* (in the State Dry Forest at *Guanica*, Puerto Rico).

*C. lucidus* is a small shrub in the Euphorbiaceae family, typically growing in the limestone hills, coastal forests in the Subtropical Dry Forest Life Zone in southwestern Puerto Rico [23]. In this study, leaves from *C. lucidus* were collected on the Ballena Trail at Guánica State Forest. Collections were made along the PR 333, up to 200 m north on both sides of the trail (Figure 1). This is an outcrop shallow soil area rich in cactus scrub type vegetation.

**Figure 2:**
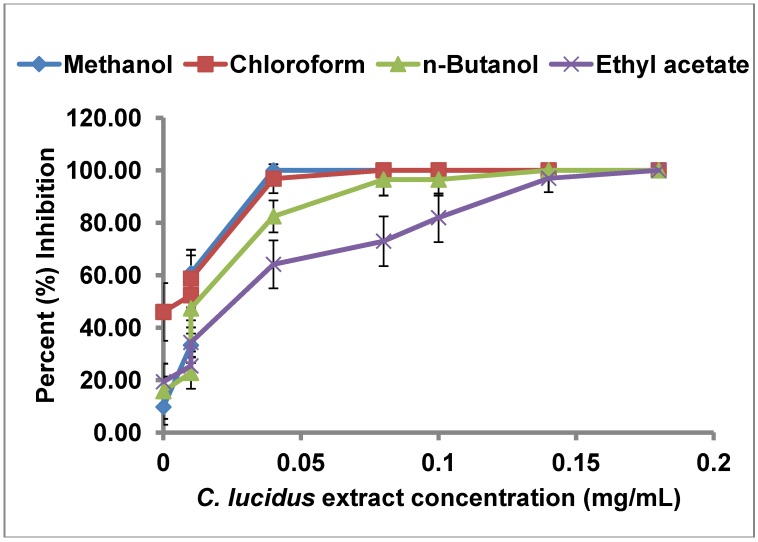
Dose-response curves of different partitions of *C. lucidus* crude leaf extract.

In our screening, the partitioned extracts from *C. lucidus* displayed the highest inhibition of uPA, and were thus investigated further for determination of their half maximal inhibitory concentrations (IC_50_). These studies were performed with the crude methanol extract and the ethyl acetate, *n*-butanol, and chloroform partitions. The petroleum ether partition was not used since it showed a weak inhibitory activity. Serial dilutions (2–200 µg/mL) of the crude methanol extract and the three partitions were prepared and tested for uPA inhibition (Figure 2). The highest concentration used (in the final analysis) was the minimal concentration that caused 100% inhibition. The data so obtained was plotted as dose response curves and linear regression performed to obtain the IC_50_ from each extract (Table 3). For all extracts, the degree of inhibition increased linearly with an increase in the extract concentration. Among the four extracts of *C. lucidus*, the chloroform extract showed the lowest IC_50_ value (3.52 µg/mL). The methanol crude extract as well as the other three extracts obtained through partitioning had IC_50_ values below 50.0 µg/mL. These results are promising since it is generally accepted that for any extract to merit chemical investigation, it must have an IC_50_ value below 100 µg/mL [24]. In a similar study Jedinak *et al*. screened methanol extracts from 30 Slovak trees for anti uPA activity. They reported that the extracts from *Acer platanoides* and *Rhustyphina* showed uPA inhibition activity with IC_50_ values of 171.1 µg/mL and 38.3 µg/mL respectively [15]. The extract partitions obtained from *C. lucidus* have clearly a higher uPA inhibitory activity as evidenced by their IC_50_ values. The presence of inhibitory activity in all the three partitions (*n*-butanol, chloroform, ethyl acetate) could be either due to presence of different uPA inhibitors in the extracts or due to different solubility of the same compound in the used solvents. In either case, the results confirmed the presence of a strong inhibitor that is highly soluble in chloroform.

**Table 3:** IC_50_ values of the extracts of leaves of *C. lucidus*.

Extract	IC_50_ (µg/mL)
Crude methanol	13.52
Chloroform partition	3.52
*n*-Butanol partition	26.51
Ethyl acetate partition	41.70

### 2.3. Selectivity of uPA Inhibitory Activity of C. lucidus Extracts

An important requisite to qualify as an ideal candidate for anti-metastatic therapy is that the uPA inhibitor must show high selectivity for uPA over other trypsin-like proteases [25,26]. A closely related enzyme, tissue-type plasminogen activator (tPA), also acts via activation of plasminogen to plasmin. However, it has not been shown to play any role in cancer metastasis, rather it is a key component of the fibrinolytic cascade and thus considered indispensable. Achievement of adequate selectivity over tPA, and other similar enzymes such as thrombin and plasmin, is therefore an important requirement in a therapeutically valuable uPA inhibitor [13]. The four *C. lucidus* extracts (0.18 mg/mL) were thus tested for inhibition of equal number of moles (0.002 nmoles) of each of the three enzymes: uPA, tPA and plasmin. The results have been summarized in Table 4. The crude methanol extract did not show any inhibition of tPA or plasmin but a 57% inhibition of uPA. Among the three partitions, the *n*-butanol partition showed highest selectivity for uPA (0% inhibition of both tPA and plasmin) followed by the chloroform partition that showed a low inhibition of both tPA and plasmin as comared to uPA. The ethyl acetate partition showed a higher inhibition of plasmin as compared to uPA and no inhibition of tPA. This indicates the presence of separate inhibitors of tPA and plasmin in the crude methanol extract at concentrations so low that no activity could be seen under tested conditions. However the fractionation of this extract with other solvents probably leads to the concentration of these inhibitors and hence the activity is observable in some of the partitions.

**Table 4:** Selectivity of the extracts of leaves of *C. lucidus*: inhibition of uPA and related enzymes.

	Percent (%) Inhibition		
	uPA	tPA	Plasmin
Crude methanol extract	57.0 ± 2.2	0.0 ± 0.0	0.0 ± 0.0
Chloroform partition	74.0 ± 2.6	19.0 ± 0.8	14.0 ± 0.9
*n*-Butanol partition	40.0 ± 1.5	0.0 ± 0.0	0.0 ± 0.0
Ethyl acetate partition	24.0 ± 3.1	0.0 ± 0.0	32.0 ± 1.7

The uPA inhibitors detected in the crude extracts of *Rhustyphina* bark by Jedinak *et al*. had higher IC50 values and were found to be broad spectrum for all tested serine proteases [15]. Thus the uPA inhibitory activities found in *C. lucidus* are promising both with respect to potency and selectivity. It can be concluded that the chloroform partition has a lower selectivity for uPA but also a lower IC50 value, and hence a higher potency, as compared to the n-butanol partition. The absolute selectivity for uPA in n-butanol partition is encouraging since it implies fewer side effects in case of clinical use.

### 2.4. Effect of C. lucidus Extracts on Cell Viability

Since the *C. lucidus* extract/partitions showed high inhibitory activity and selectivity for uPA, the MTS assay was performed using serial dilutions of each of the four extracts (0.01–1.0 mg/mL), to further confirm if these extracts actually exhibited any cytostatic activity *in vitro*. PaCa-2, a pancreatic carcinoma cell line, was used for this study. Known quantities of the extracts were dried under N_2_ gas and then suspended in DMSO and finally redissolved in the cell culture media which was then added to the microwell plates containing viable cells. DMSO accounted for 1% (v/v) of the final volume of the extract containing media, and did not affect cell viability as evidenced by culturing cells in media containing the same amount of DMSO (1% v/v) but no extracts.

**Figure 3:**
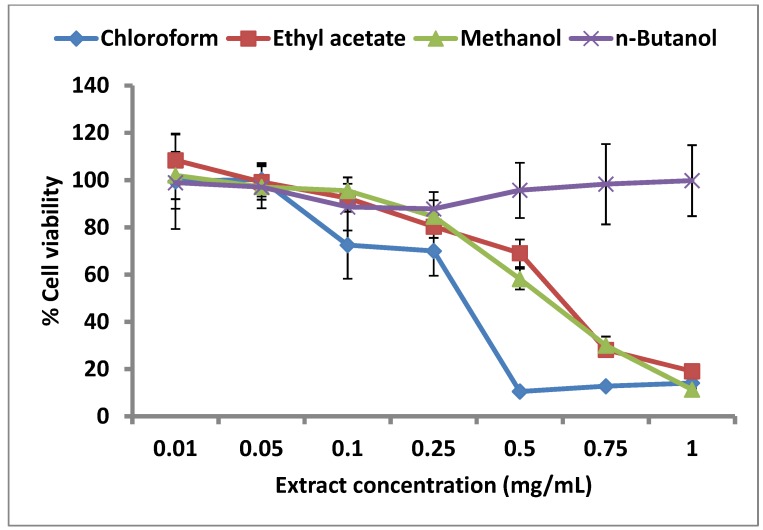
Cytostatic effect of *C. lucidus* extract on PaCa-2 cells.

The effect of each of the four extracts on cell viability has been presented in Figure 3. The chloroform partition clearly showed the strongest cytostatic effect on the proliferation of human pancreatic carcinoma cells (PaCa-2). Cell viability decreased sharply until the extract concentration of 0.5 mg/mL, but no further decrease was seen at higher chloroform partition concentrations. While crude methanol extract and ethyl acetate partition showed comparable negative impact on cell proliferation, the *n*-butanol partition seemed to decrease cell viability until the extract concentration of 0.25 mg/mL, beyond which erratic results (high standard deviations) were obtained. The decrease in cell viability seen in the case of methanol and ethyl acetate extracts had an obvious inverse linear dependence on the extract concentration in the entire range tested. This linear dependence could not be seen in case of *n*-butanol and chloroform partitions beyond certain concentrations, which might be due to the fact that higher concentrations of each of the two partitions were not completely soluble in cell culture media even after addition of the maximum possible amount of DMSO without compromising cell viability. While in case of chloroform, linearity could be obtained until the conc. of 0.50 mg/mL, the *n*-butanol partition was relatively insoluble at concentrations higher than 0.1 mg/mL (Figure 3). The limited solubilities of chloroform and *n*-butanol partitions can be explained on the basis of respective solvent polarities: *n*-butanol (4.0), chloroform (4.1), ethyl acetate (4.4), methanol (5.1). The lowest polarity of *n*-butanol would lead to the partitioning of most hydrophobic compounds into this solvent and hence their insolubility in the aqueous cell culture media. chloroform is only slightly more polar than *n*-butanol, thus explaining the limited solubility of these and also the chloroform partitioned compounds in the aqueous media.

However, the cytostatic effect of the chloroform partition was clearly stronger than the other three partitions, which correlates with the results obtained from the uPA inhibition assays, where the same extract showed also the lowest IC_50_. Since this study was performed with crude extracts, it cannot be concluded that the compounds causing uPA inhibition are the same as the ones responsible for cytostatic activity; however, in view of the well-established role of uPA in cancer metastasis, it may be hypothesized that the cytostatic effect of the *C. lucidus* extracts is caused by their uPA inhibitory activity.

## 3. Experimental

### 3.1. Materials

Urokinase type plasminogen activator (uPA), fibrinogen, thrombin, plasminogen, plasmin, bovine serum albumin (BSA), and organic solvents were obtained from Sigma-Aldrich Co. (St. Louis, MO, USA). The tPA was a generous donation from Genentech (Genentech Inc., CA, USA). Fetal bovine serum (FBS) was obtained from Thermo Scientific Hyclone (Logan, UT, USA). PaCa-2 cell line was obtained from ATCC (Manassas, VA, USA). Cell culture media, penicillin, streptomycin, optiMEM buffer, phosphate buffered saline (PBS), trypsin and trypan blue, were all purchased from Invitrogen (Frederick, MD, USA). All chemicals were of analytical grade purity and were used without further purification. Deionized water was used for the preparation of all solutions. The MTS kit was purchased from Promega (Madison, WI, USA).

### 3.2. Collection of Plant Materials

The leaves of 14 different plant species were collected on PR-333, Ballena Trail, Guanica State Forest, Puerto Rico. The location is 17°57'30.88" N, 66°51'42.76" W. The plant samples were identified and authenticated by Dr. Augusto Carvajal at the Department of Biology (University of Puerto Rico-Cayey). The collection voucher (collection number: 16OCT2010-1) was deposited in the herbarium of University of Puerto Rico at Rio Piedras.

### 3.3. Preparation of Extracts

The collected samples were washed with water and dried to a constant weight in an oven at 40–50 °C. The dried samples were ground to fine powder using a laboratory grinder. The material (20 g) from each plant was extracted with methanol (100 mL) for 24 h at room temperature. The extracts were dried under pressure using a rotary evaporator. All the dried extracts were resuspended in deionized water and tested for inhibition of uPA (as described in Section 3.4). The extracts that tested positive were then partitioned with petroleum ether, chloroform, ethyl acetate and *n*-butanol in a serial manner using a separatory funnel. These partitioned extracts were dried using a rotary evaporator. All dried extracts were then stored in air-tight bottles at 4 °C until used for the bioactivity testing.

### 3.4. Bioassay of uPA-Inhibitory Activity of Extracts

All the dried extract partitions were re-resuspended in methanol to a final concentration of 1.0 g/mL and stored in amber glass bottles. Methanol was used to prepare the extracts for bioassay as previous experiments in our laboratory have shown that methanol does not have any observable adverse effect on uPA activity at the concentrations used. The inhibitory effect of extracts on uPA activity was assayed using a slightly modified fibrin plate method [27]. The fibrin plate was prepared by solidifying a solution consisting of: 1% agar (w/v) in 20 mM Tris-HCl buffer containing 1.07 M NaCl, thrombin (0.02 NIH unit/μL), fibrinogen (0.024% w/v), and BSA (0.1% w/v). Five wells were then created in each plate, where in, each well could hold up to a maximum of 20 µL of the sample. The plates were stored at 4 °C until the samples were ready to be tested.

**Figure 4:**
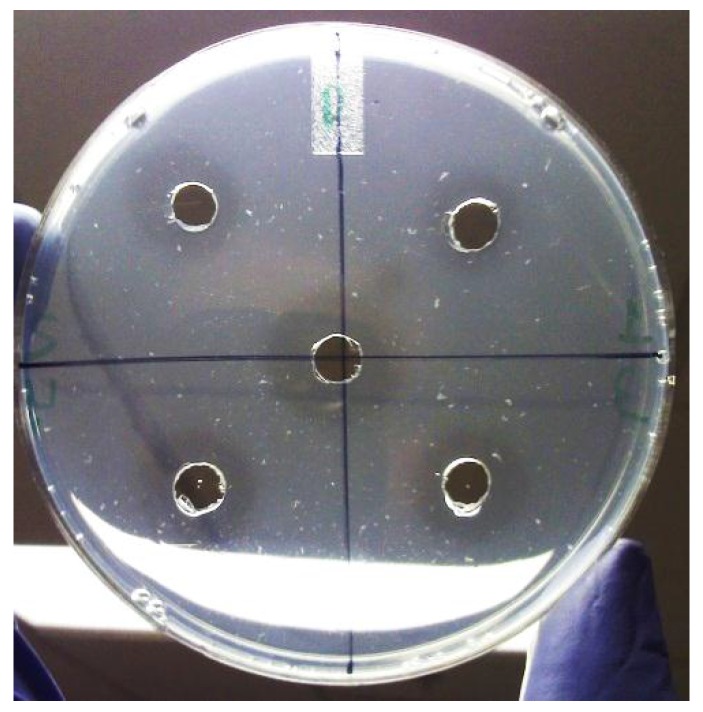
Fibrin plate assay for screening of the plant extract for presence of uPA inhibitory activity.

uPA (from human urine, Sigma) was used as the positive control as well as the target enzyme for testing the inhibition potential of the extracts. A known quantity of uPA was incubated with plant extracts for 30 min at 37 °C. Plasminogen was then added to the uPA-extract mix and incubated at 37 °C for another 15 min. Twenty µL of each sample mixture were then pipetted into the respective well in the fibrin plate. The plates were incubated at 37 °C for up to 24 h. uPA activity appeared as clear zones of lysis around the wells, and sizes of zones were measured at 12 h and 24 h of incubation (Figure 4). The positive control consisted of uPA incubated with pure methanol and plasminogen to account for any possible enzyme denaturation by the solvent. Plasminogen (without any uPA) incubated with methanol was used as the negative control. All experiments were done in triplicate.

### 3.5. Determination of IC_50_

Dose-response curves were obtained by performing the fibrin plate assay for serial dilutions of the partitioned extracts in a concentration range from 2 to 200 µg/mL using MET as the solvent. The half maximal inhibitory concentrations (IC_50_) for uPA inhibition were determined from these dose-response curves after linear regression analysis [20]. All experiments were done in triplicate.

### 3.6. Bioassay of uPA Selectivity of Extracts

To determine if the extracts were selective for uPA, or affected other closely related proteases too, the fibrin plate assay was performed to check the potential of the extracts to inhibit tPA and plasmin. Equal moles (0.002 nmoles) of tPA, uPA, and plasmin were used in these studies, while *C. lucidus* partitions were used at a concentration of 0.18 mg/mL.

#### 3.6.1. Inhibition of tPA

The fibrin plate assay was performed as described for uPA, with the difference that uPA was replaced with tPA (Genentech). All experiments were done in triplicate.

#### 3.6.2. Inhibition of Plasmin

A known quantity (0.002 nmoles) of plasmin (Sigma) was incubated with plant extracts for 30 min at 37 °C. Twenty µL of each sample mixture were then pipetted into the respective well in the fibrin plate. The plates were then incubated at 37 °C for up to 24 h. Plasmin activity appeared as clear zones of lysis around the wells and sizes of zones were measured at 12 h and 24 h of incubation. The positive control consisted of Plasmin incubated with pure methanol to account for any possible enzyme denaturation by the solvent. The extract mixed with phosphate buffered saline (PBS) was used as the negative control. All experiments were done in triplicate. The % inhibition was calculated as the % decrease in enzyme activity in the presence of the extracts as compared to the enzyme activity in the absence of extract.

### 3.7. Cell Culture Assays

These experiments were carried out using PaCa-2 cells. PaCa-2 cells were grown in MEM medium with L-glutamine supplemented with 10% FBS and 1% penicillin. The cells were incubated at 37 °C and 5% CO_2_.

*MTS Assay*: For determining the effects of *C. lucidus* extracts on cell viability, the 3-(4,5-dimethylthiazol-2-yl)-5-(3-carboxymethoxyphenyl)-2-(4-sulfophenyl)-2H-tetrazolium (MTS) assay was employed. Cells (4 × 10^4^ cells/well) were seeded into 96-well microtiter plates (100 μL of penicillin free culture medium with 10% FBS). After 24 h, culture media were replaced with culture media containing serial dilutions of the extracts (0.01–1.0 mg/mL, pre-suspended in DMSO (1% v/v)), and the cells were incubated for 24 h. At the end of this period, 20 μL of MTS was added to each well. After 2 h, the optical intensity of each was measured spectrophotometrically at a wavelength of 490 nm in a microplate reader ((Abs)_treated_). The spectrophotometer baseline was calibrated using culture medium containing the respective extract but without cells. The relative cell viability was calculated with untreated cells (cultured in media containing DMSO (1% v/v) as a control ((Abs)_non-treated_) using the following equation:

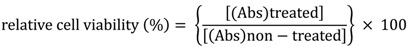
(1)

## 4. Conclusions

This study successfully demonstrated the presence of inhibitors of uPA in the leaves of *C. lucidus*. The inhibitory activity was strongest in the chloroform partition, with an IC_50_ value of 3.52 µg/mL. The same partition also showed highest cytostatic activity over PaCa-2 cells. A moderate to high selectivity for uPA was also observed in the different partitions, with the *n*-butanol partition showing the highest selectivity. The isolation and structural elucidation of this (these) uPA inhibitor(s) could lead to promising drug candidate(s) for prevention of cancer metastasis. To the best of our knowledge, this is the first report of presence of uPA inhibitor(s) in *C. lucidus*. *C. lucidus* leaves’ extract is thus a good candidate for further activity-monitored fractionation to identify the active principle(s).

## Abbreviations

uPA, urokinase type plasminogen activator; *C. lucidus*, *Croton lucidus*; IC50, inhibitory concentration 50; MTS, 3-(4,5-dimethylthiazol-2-yl)-5-(3-carboxymethoxyphenyl)-2-(4-sulfophenyl)-2H-tetrazolium; FBS, fetal bovine serum; tPA, tissue type plasminogen activator.
